# Advances in the Supramolecular Chemistry of Tetracoordinate Boron-Containing Organic Molecules into Organogels and Mesogens

**DOI:** 10.3389/fchem.2021.708854

**Published:** 2021-09-07

**Authors:** Sanchita Shah, Parvati Marandi, P. P. Neelakandan

**Affiliations:** Energy and Environment Unit, Institute of Nano Science and Technology, Mohali, India

**Keywords:** luminescece, self-assembly, boron, gels, liquid crystals

## Abstract

Boron-containing organic compounds are well accepted as a class of compounds having excellent photophysical properties. In addition to the unique photophysical properties, the ease of synthesis and structural robustness make tetracoordinate boron complexes ideal for a variety of applications. While significant light has been thrown on their luminescence properties, there is no collective attention to their supramolecular chemistry. In this mini review, we discuss the progress made in the supramolecular chemistry of these compounds which includes their utility as building blocks for liquid crystalline materials and gels largely driven by various non-covalent interactions like H-bonding, CH-π interactions, BF-π interactions and Van der Waals forces. The organoboron compounds presented here are prepared from easy-to-synthesize chelating units such as imines, diiminates, ketoiminates and diketonates. Moreover, the presence of heteroatoms such as nitrogen, oxygen and sulfur, and the presence of aromatic rings facilitate non-covalent interactions which not only favor their formation but also helps to stabilize the self-assembled structures.

## Introduction

Organoboron compounds are typically employed in organic chemistry as reagents and catalysts. However, boron has emerged as a key element for organic luminescent materials in the recent past. Several tri- and tetra-coordinated boron containing organic compounds have been synthesized and were demonstrated to have excellent photophysical properties (Loudet and Burgess, 2007; Li et al., 2013; Frath et al., 2014; Mukherjee and Thilagar, 2016). Properties such as environment sensitive luminescence, long lifetimes and high carrier mobility has allowed the usage of boron-containing organic compounds as organic light-emitting diodes, organic field-effect transistors, photoresponsive materials, photosensitizers, sensors and imaging materials (K. Tanaka and Chujo, 2012; Anthony, 2012; Awuah and You, 2012; Rao and Wang, 2011; Jäkle, 2010; Entwistle and Marder, 2002).

The delocalization of the π-electrons of organic chelates to the vacant *p*-orbitals of boron rigidifies the boron-containing organic compounds and thus stabilizes the π-conjugated skeletons (Shah et al., 2018). Such ring-fused structures not only constrain the π-conjugated framework to intensify the emission but also lower the energy levels of the lowest unoccupied molecular orbital. The type of the ligands and the nature of the substituted groups on either the ligands or boron have a great influence on the photophysical properties of these compounds. A variety of ligands have been investigated as boron coordination motifs and the fluorescence of the resulting organoboron compounds cover a wide spectral range in the UV and visible region. Several reviews have been published that compile the optical properties and applications of different classes of boron-containing organic molecules (Boens et al., 2012; Kamkaew et al., 2013; Li et al., 2013; Maeda and Bando, 2013; Frath et al., 2014; Mukherjee and Thilagar, 2016; Chen et al., 2017b; Matsuoka and Nabeshima, 2018).

Supramolecular chemistry of the organoboron compounds pertains to their usage as chemosensors, inclusion compounds, liquid crystalline materials, gels and luminescent materials. Among the various classes of self-assembled structures, gels and liquid crystals are interesting because their nanostructure and properties can be tuned by subtle modifications in the chemical composition. The formation of both gels and mesogens is driven by the balance between various non-covalent interactions such as hydrogen bonding, electrostatic interactions, hydrophobic forces, CH-π interactions, BF-π interactions and Van der Waals forces. The presence of hetero atoms in boron-containing organic compounds along with the π-conjugated core offers a stable platform for the formation of gels and mesogens. Although their supramolecular chemistry is as intriguing as their luminescence properties, their self-assembly properties have not been reviewed extensively. This mini-review focusses on the supramolecular assemblies such as gels and liquid crystals formed by tetra-coordinate boron-containing organic molecules containing chelates such as imines, diiminates, ketoiminates and diketonates. Boron-dipyrromethenes (BODIPYs) are the most famous members of the organoboron family. But, as there are several reviews that summarize the developments in the chemistry of BODIPYs (Loudet and Burgess, 2007; Galbraith and James, 2010; Boens et al., 2012; Kamkaew et al., 2013; Lu et al., 2014; Cherumukkil et al., 2018), we do not intent to discuss any BODIPY compounds. We have subdivided the mini-review into two sections namely gels and mesogens with one figure representing each section.

## Self-Assembly into Gels

**FIGURE 1:**
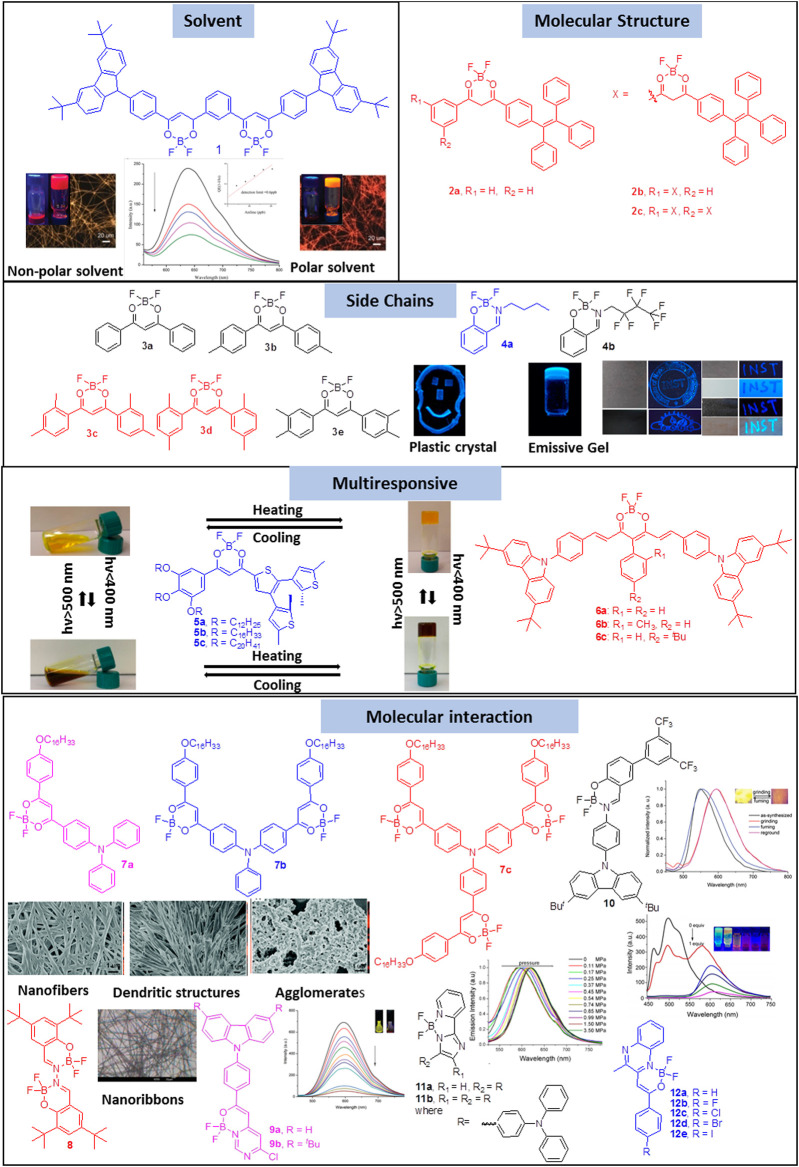
Gelation of boron-difluoride compounds on the basis of the effect of solvent, molecular structure, effect of substituents and intermolecular interactions. Reproduced with permission from Sun et al., 2018, copyright 2018 Royal Society of Chemistry. Reproduced from Naim et al., 2021, copyright 2021 American Chemical Society. Reproduced from Wong et al., 2017, copyright 2017 American Chemical Society. Reproduced with permission from Qian et al., 2015, copyright 2015 Royal Society of Chemistry. Reproduced with permission from Gong et al., 2015, copyright 2015 Royal Society of Chemistry. Reproduced with permission from Mi et al., 2018, copyright 2018 Royal Society of Chemistry. Reproduced with permission from Sun et al., 2017, copyright 2017 Royal Society of Chemistry. Reproduced with permission from Wang et al., 2017, copyright 2017 WILEY-VCH. Reproduced with permission from Wu et al., 2017, copyright 2017 WILEY-VCH.

Organogels are semisolids composed of a gelling molecule organized into a continuous network *via* physical or chemical crosslinking that exhibits no flow in the steady state. The formation of organogels depends on various factors such as the nature of the gelator, solvent, temperature and the time for gelation. Organogels are widely employed as matrices for inks, paints and cosmetics, and their stimuli-responsive properties allow them to be used for applications wherein sustained and controlled release is necessary (Abdallah and Weiss, 2000; Varaprasad et al., 2017; Crump et al., 2021; Das et al., 2021; Esposito and Kirilov, 2021). Boron-difluoride complexes are emerging as an important class of organogelators because they can promote non-covalent interactions through the hetero atoms that are present inherently in their structure. Moreover, these compounds are easy to synthesize and functionalization with traditional gelation-inducing moieties could be circumvented. Further, their excellent stability and photophysical properties such as high fluorescence in solution and solid state, high electron affinity and large molar extinction coefficients make them excellent building blocks for gelation. In this mini review, we have classified the gelation of boron-difluoride compounds on the basis of the effect of solvent, molecular structure, effect of substituents and intermolecular interactions (Figure 1).

One of the crucial factors for gelation is the ability of the gelator to trap solvent molecules. The balance between the interaction of the gelator with itself and with the solvent is determined by the solvent as solvent plays a critical role in the nucleation and growth processes of self-assembly. Sun et al. showed that the polarity of the solvent affects the gelation properties of β-diketonate difluoroboron complex **1** functionalized with *tert*-butyl carbazole moiety (Sun et al., 2018). Complex 1 formed two different colored organogels: a red gel was formed in low polar solvent mixtures such as *o*-dichlorobenzene/cyclohexane (v/v = 1/5) whereas an orange gel was formed in high polar solvent mixtures like *o*-dichlorobenzene/cyclohexane (v/v = 1/2). The difference in the color of the gels were attributed to the formation of *J*- and *H*-aggregates in these solvent mixtures which resulted in the formation of twisted and straight nanofibers, respectively. Further, xerogel based films of complex 1 were found to be sensitive towards n-propylamine and aniline with a detection limit of 1 ppb and 0.6 ppb respectively.

In addition to solvent, chemical composition of the molecule also plays a vital role in the self-assembly pattern of tetra coordinated difluoroboron complexes. Zhai et al. illustrated that a boomerang shaped β-diketone difluoroboron complex without traditional gelation groups forms organogels with the introduction of tetraphenylethylene unit 2a-c (Zhai et al., 2017). The xerogel of 2b obtained from *p*-xylene formed nanorods with strong orange red emission having diameters in the range of 0.67–1.78 μm due to the favorable boomerang shaped π-conjugated skeleton that facilitated self-assembly whereas 2a and 2c did not form gel. Furthermore, XRD measurements indicated that molecules of 2b were packed in an anti-parallel bimolecular layered structure in the gel state. It was interesting to observe that the emission of 2 in xerogel-based film was quenched significantly in the presence of triethylamine vapors.

Similar to the molecular structure, appropriately placed substituents also exert a significant influence on the self-assembly and the gelation properties of the tetra coordinated boron difluoride complexes. Zhu et al. demonstrated the importance of methyl group in the gelation of β-diketone difluoroboron complexes 3a–e (Zhu et al., 2020). The self-assembly of these complexes were studied in various solvents like hexane, cyclohexane, petroleum ether and toluene. Xerogels 3c and 3d were observed to form flake-like lamellar structures with porous networks which implied that a large volume of solvent was entrapped in the gel state. The gels were also found to be super-hydrophobic with water contact angles ranging from 145–153°. Detailed investigation into the self-assembly pattern revealed that the number and position of methyl groups played a vital role in the self-assembly. Intermolecular π-π stacking and non-covalent bonding between methyl groups and the neighboring molecules were ascertained to be the driving forces for gelation. Our group recently showed that the chemical composition of the sidechain has a significant influence on the self-assembly as well as the mechanical and luminescent properties of boron difluoride complexes (Naim et al., 2021). While complex 4a with an alkyl chain formed plastic crystals whereas the complex 4b with a fluoroalkyl chain favored the formation of organogels. Based on crystallography and NMR data, it was established that the ability of 4a and 4b to form strong and weak C−H···F and B−F···π interactions, respectively, was the key in defining the different self-assembly behavior. It was also demonstrated that 4b could be used as a fluorescent security marker on a variety of surfaces such as paper, granite and wood.

A combination of the above mentioned effects, *viz*. the effect of the solvent, molecular structure and substituents, can lead to organogels with multi-responsive properties. Wong et al. reported a few β-diketonate boron complexes (5) that functioned as photo-responsive organogelators with photo-switchable behavior (Wong et al., 2017). Among the different compounds, only 5c with a long alkoxy chain formed gels with fibrous structure in toluene and benzene. The gel of 5c in benzene exhibited green luminescence while in 1,4-dioxane, a bluish-green luminescence was observed. Moreover on heating the benzene/toluene gels of 5c, a color change was observed from orange to yellow because of the photocyclization of the dithienylethylene moiety. Zhai et al. reported a few β-diketone difluoroboron complexes that formed luminescent gels *via* self-assembly into one-dimensional nanorods (Zhai et al., 2019). Detailed investigations revealed that π-π interactions were the main driving force for the molecules to pack into parallel layered structures in the gel state. Organogels of the compound 6c were emissive in the red region and their xerogels were observed to be sensitive towards aniline with a detection limit of 2 ppb.

The presence of aromatic core that favors π-π interactions is another factor that contributes to gelation. The directionality and rigidity of these interactions are vital in deciding the spatial arrangements of the molecules during gelation. Several strategies have been developed to tune the strength of π-π interactions by elongating the conjugation or by appropriately introducing functional groups. Qian et al. showed that molecular structure as well as balanced π-π interactions play a vital role in the gelation property exhibited by β-diketones difluoroboron complexes. In a series of triphenylamine functionalized β-diketones difluoroboron complexes **7**, it was reported that the asymmetric complex 7a exhibited a better gelation property in comparison to the symmetric complexes 7b and 7c (Qian et al., 2015). The difference in the self-assembly behavior was explained on the basis of the formation of different types of nanostructures. While 7a self-assembled into a three-dimensional network of straight nanofibers, 7b self-assembled into numerous nanofibers forming dendritic structures. On the other hand, 7c self-assembled into three-dimensional network of ill-defined agglomerates. Similarly, Gong et al. showed that salicylaldehyde-hydrazone based β-ketoiminatedifluoroboron complexes 8 self-assembled into nanoribbon-like features resulting in the formation of gels with intense blue emission (Gong et al., 2015). In the complex 8 the non-planar aromatic unit experienced large steric hindrance from the *tert*-butyl group thereby decreasing the strength of π-π interactions thereby inducing gelation. On the other hand, on further extending the conjugation no gel formation was observed due to an imbalance in π-π interactions. Another similar report by Mi et al. showed that the pyrimidine containing β-iminoenolate-difluoroboron complexes 9a–b self-assembled into organogels upon the introduction of *tert*-butyl group which provided an optimum strength to π-π interactions (Mi et al., 2018). The xerogel-based film of 9b was observed to emit yellow light and was used as a fluorescent sensor to detect vapors of trifluoroacetic acid with a decay time and detection limit of 0.8 s and 260 ppb, respectively.

Sun et al. showed that both *tert*-butyl and trifluoromethyl groups are important in the formation of organogels in the case of carbazole based salicylideneimine-boron complexes 10 (Sun et al., 2017). Only 10 containing both the *tert*-butyl and trifluoromethyl group formed gels with a three dimensional network of intertwined nanofibers. These nanofibres were also observed to be emissive due to the formation of *J*-aggregates in the gel state and exhibited reversible piezofluorochromic behavior. Another gelator based on balanced π-π interactions was reported by Wang et al. which consisted of triphenylamine-functionalized boron complexes 11a and 11b containing 2-(2′-pyridyl)imidazole moieties that self-assembled into gels and showed piezochromic property wherein dried gel exhibited better sensitivity to pressure as compared to the regular crystalline powder (Wang et al., 2017). Using a series of halogenated β-iminoenolate difluoroboron complexes 12a-h, Wu et al. showed that not only the conjugation but the electronic effect also plays a vital role in the gelation abilities (Wu et al., 2017). It was observed that for excellent gelation, strong π-π, CH···F, and CH···Br interactions played a key role. Interestingly, 12c-e were found to be emissive not only in solution but also in organogels and xerogel based films. The nanofiber films of 12d were sensitive towards trifluoroacetic acid with a decay time and detection limit of 0.5 s and 0.17 ppm, respectively.

## Self-Assembly into Mesogens

Liquid crystals (LCs) have been long known from 1888 which grew into a multibillion dollar industry over the time (Mitov, 2014). LCs can be defined as a state of matter characterized by the regularity in the periodic arrangement of atoms/molecules as in solids as well as the anisotropy as in liquids. They display a unique blend of the long range order and mobility. Materials that display liquid crystal phases are called mesogens. LCs are typically formed by calamitic (rod-like), discotic (disc-shaped) or bent core (banana-shaped) molecules. One major class of LCs is the thermotropic LCs which have the ability to display mesomorphic behavior as a function of temperature and as a result the molecules/atoms self-assemble into three different kinds of arrangements: namely nematic, smectic and chlolesteric. In nematic phase, molecules are aligned with the director axis parallel to each other, but there is no positional order. When these molecules further organize into layered arrangements stacking into two dimensional manner, smectic phase is formed which display a degree of translational order not present in nematic phases. Cholesteric LCs have the director axis spiraling around with a pitch, these are mainly the nematic mesogens that consist of a chiral center. Columnar mesophase fall into another category which are represented by stacked columns of molecules packed together to form an array. They are shaped liked disks instead of long rods. As LCs are responsive to various stimuli, their molecular order rapidly responds to environmental factors like temperature, electric fields, magnetic fields or chemical adsorption. While LCs are immensely popular for their application in flat panel displays, their special properties have been used in a number of other applications as well such as organic electronics, nanoparticle organization, LC colloids, LC elastomer actuators, and chemical and biological sensors. Apart from these, liquid crystals have also found utility in thin-film thermometers and switchable display windows (Lagerwall and Scalia, 2012; Iino et al., 2015; Urbanski et al., 2017; Chen et al., 2021).

**FIGURE 2:**
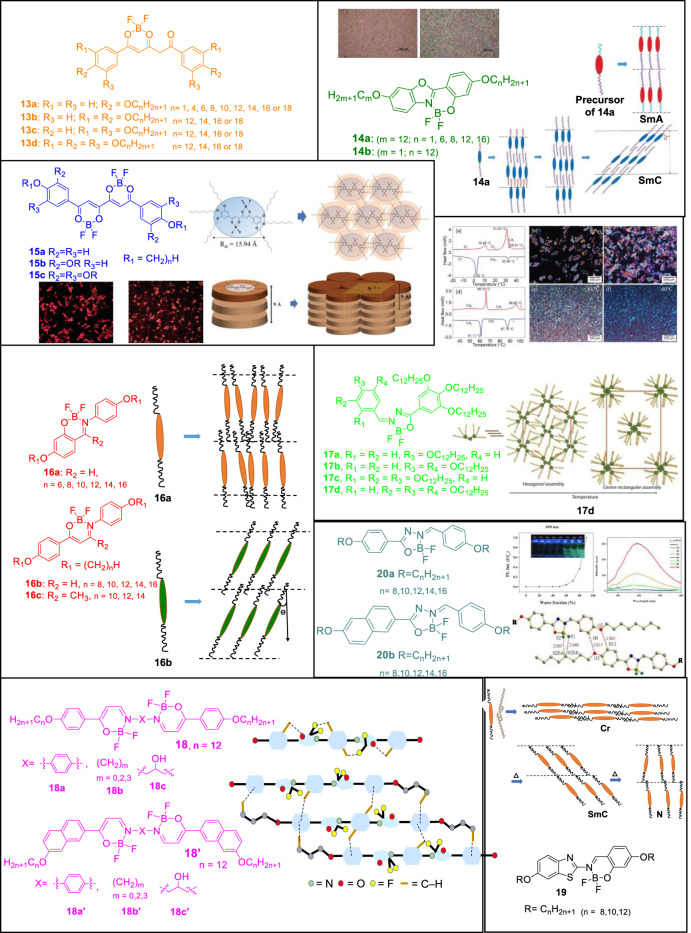
Chemical structures and schematic representation of boron-difluoride compounds self-assembling into mesogens. Reproduced with permission from Chen et al., 2017a, copyright 2017 Royal Society of Chemistry. Reproduced with permission from Chen et al., 2017b, copyright 2017 Royal Society of Chemistry. Reproduced with permission from R. Vinayakumara et al., 2019, copyright 2019 Royal Society of Chemistry. Reproduced with permission from Cai et al., 2021, copyright 2021 Royal Society of Chemistry.

Boron-containing organic molecules have been studied for their mesogenic properties along with luminescence properties (Camerel et al., 2007; Maeda et al., 2010; Olivier et al., 2010; Sánchez et al., 2010; Turanova et al., 2010; Benstead et al., 2011a, 2011b; Maeda et al., 2011; Olivier et al., 2012; Bando et al., 2013; Giziroglu et al., 2014; Sánchez et al., 2014; Fang et al., 2017; Feng et al., 2018; Xiong et al., 2018; Cheng et al., 2019; Liu et al., 2019). The very first reports were put forward by Turanova et al. wherein the boron difluoride-diketonate complex obtained from a non-mesogenic diketonate exhibited liquid crystalline properties along with photoluminescent properties (Turanova et al., 2006). In concurrence with the previous section, this mini-review will focus on the advances made using diketonates and ketoiminates in the past few years (Figure 2).

Sánchez et al. reported alkyloxyphenyl-substituted β,δ-triketonatedifluoroboron complexes 13 wherein the presence of varying alkyl chains in each aromatic group of the triketonate ligand dictated the mesomorphic behavior of the compound (Sánchez et al., 2016). Smectic C mesophases were found for compounds carrying two lateral chains whereas those consisting of four or six chains led to discotic lamellar and hexagonal columnar mesophases, respectively. Compounds 13a with long-tailed substituents (n = 16, 18) showed liquid crystal behavior, while derivatives with shorter alkyl chains (n < 14) showed crystalline polymorphism and a direct melt to isotropic liquid. Contrary to previous reports on non-mesogenic monocatenar symmetrically alkoxyphenyl substituted β-diketonate containing difluoroboron complexes, the reported long chain monocatenar aryl-substituted triketonate derivatives showed liquid crystal properties. This was presumably due to an appropriate length-to-width molecular ratio, which is favored with long chains and extensive conjugation in triketones. Presence of the large and rigid molecular core with extended π conjugation and molecular polarization effectively induced mesomorphism in the difluoroboron complexes. While molecular asymmetry with respect to the length of chains and the presence of varying number of alkyl chains at both phenyl rings was a prerequisite to achieve mesomorphism in β-diketonates, this requirement was not mandatory when using β,δ-triketonate ligands for BF_2_ coordination in which liquid crystal behavior was achieved even at room temperature. Compounds containing one chain or two adjacent chains in each phenyl group exhibited smectogenic behavior stemming clearly from the rod-like molecular shape. In contrast, disk-shaped complexes were obtained when two alternate or three alkyl chains were attached to the aromatic system. Consequently the complexes form lamellar discotic or hexagonal columnar mesophases, respectively.

Chen et al. further reported the liquid crystalline properties of the benzoxazoles derived boron complexes 14 (Chen et al., 2017a). The mesomorphic behavior of complexes 14 was altered upon complexation to boron difluoride and the clearing temperatures increased than the uncoordinated benzoxazoles. While the precursor benzoxazoles formed monotropic Smectic A phases, the corresponding boron difluoride complexes 14a (n = 6, 8, 12, 16) exhibited monotropic Smectic C mesophases. Molecules in the 14b category was not found to be mesogenic owing to the presence of the methoxy group which was assumed as not being polar enough to induce a stable mesophase. Intermolecular F···H bonds as observed in the crystal lattices of 14a (m = 12, n = 8) facilitated the molecules to align in head-to-head arrangements which is one of the requirements for the induction of mesophases.

Coordination to boron difluoride plays an interesting role towards appearance of liquid crystal properties as tetrahedral geometry allows a dipole strong enough to induce the mesophase. But at the same time the small size of BF_2_ does not interrupt the molecular arrangement of the mesophases. Otherwise generally, a tetrahedral or bulky core does not favor the formation of mesophases. Along with weak H bonding interactions, the lone-pair of electrons located on the two fluorine atoms also repelled other atoms intra- and intermolecularly, facilitating the weaker interactions required for the formation of kinetically stable mesophases. Chen et al. also reported a series of bis(boron difluoride) complexes derived from the corresponding substituted tetraketonates (Chen et al., 2017b). While compounds 15a and 15b carrying varying alkyl lengths were not mesogenic, compounds 15c (n = 8, 10, 12) exhibited enantiotropic columnar mesophases where a single disc-shaped molecule was stacked within columns in hexagonal columnar phases. While the precursors of 15a were mesogenic forming enantiotropic nematic or nematic/smectic C phases, the precursors of 15b-c formed crystalline phases because the overall molecular shapes of these two compounds were more likely catenar shaped, instead of linear or disc-shaped. It is noteworthy that most of the known α,β-diketonates were not mesogenic due to their bent structures which do not favor mesophases formation. A more linear core in the tetraketonate structure and a small dihedral angle of ∼1.564° resulted in the induction of mesophases compared to other non-mesogenic α,β-diketonates.

Lei et al. further reported BF_2_ complexes 16 obtained from substituted salicylideneamine and β-enaminoketonates (Lei et al., 2018). Four complexes 16a (n = 8, 10, 12, 14) showed monotropic mesogenic behavior, whereas the other two derivatives (n = 6, 16) were not mesogenic. 16b (n = 8, 10, 12, 14, 16) exhibited enantiotropic Smectic C phases and the better mesomorphic behavior of 16b was attributed to being a better rod/linear shaped molecule owing to the presence of β-enaminoketonate moiety in 16b than that of salicylideneamine in 16a. Also β-enaminoketonate 16b has more extended conjugation length than the salicylideneamine moiety in 16a. The imine moiety here not only played the role of linking group but also carried a dipole moment that enhanced the thermal stability of mesophases. An improved core planarity resulting from various weak intra- and intermolecular H-bonds facilitated a more ordered arrangement in mesogenic states. In the crystal structure, along with the H-bonding interactions, the intermolecular F···H interactions were found to be crucial, which facilitated molecules to align in a head-to-head manner. While only one complex 16c (n = 12) showed monotropic mesomorphic behavior, the other two complexes in 16c were not mesogenic as both -BF_2_ and -CH_3_ could have slightly twisted the layer packing in the crystal and liquid crystalline states. Large dihedral angle of 81.3° between the two phenyl rings of 16c was supposed to be the main reason behind the lack of liquid crystallinity.

Vinayakumara et al. reported benzylidenehydrazones substituted BF_2_ complexes 17 wherein the precursor compounds benzylidenehydrazones exhibited a columnar mesophase with a hexagonal structure which could be attributed to co-operative intermolecular hydrogen bonding (R. Vinayakumara et al., 2019). Their corresponding BF_2_ complexes showed thermotropic phase behavior. The number and position of chains around the aromatic core greatly affected the mesomorphic properties of these hemi-discoidal boron complexes. As a result, isomeric complexes showed different thermotropic phase behavior and exhibited distinct columnar mesophase at different temperature ranges. While the alkyl chains around the planar core moiety helped to acquire disc-shaped molecules, the central core-system consisting of three rings adopted an entirely planar geometry which supported the stabilization of the columnar arrangement.

Lei et al. derived liquid crystals of bis(BF_2_) complexes 18 from Schiff base β-enaminoketones (Lei et al., 2020). Mesomorphic properties were observed to be dependent on the structure of the central spacer and terminal groups and most of the synthesized molecules formed SmC or SmA phase except complexes 18b (m = 2, 3; n = 12) and 18c (n = 12). The structure of 18a was linear enough to be considered as rod-shaped which favored the layered smectic arrangement. While compound 18b wherein m = 0 was purely mesogenic, the other two compounds 18b (m = 2, 3) showed crystalline phases and the absence of mesogenic behavior for derivatives with m = 2 and 3 was attributed to the more flexible ethylene and propylene spacer units. Upon incorporation of ethylene or propylene group in compounds 18b (n = 12), all clearing temperatures were significantly lowered. Also, the mesogenic behavior did not improve upon introduction of a hydroxyl group in the C2-atom of compound 18c as a bulky-OH group incorporated closer to core center could not favor the molecules to pack in the solid or liquid crystal state. Introduction of a terminal naphthalenyl moiety significantly improved the mesomorphic properties and thermal stabilities of all four compounds 18′ as compared to their homologues 18. Compound 18c’ (n = 12) was truly mesogenic and its improved mesomorphic behavior from 18c was attributed to more balanced intermolecular π-π interactions by terminal naphthalenyl ring and hydrogen bonding by central hydroxy-OH group. “Shape-effect” of the compound 18c′ also had a role to play along with an appropriate aspect ratio necessary for the induction of the mesophases. Elongation of the rigid molecular core also resulted in broadening of the mesophases. Boron complexes 18′ with terminal naphthalenyl ring were found to be more stable in higher temperatures than 18.

Hsu et al. reported boron difluoride complexes 19 from benzothiazoles that formed nematic and smectic phases as is common for rod shaped molecules (Hsu et al., 2020). In the crystal lattice, all alkoxy chains were slightly interdigitated, giving a smaller layer distance both in N and SmC phases upon heating. The intramolecular H-bonds in the benzothiazole moiety kept the phenolic ring nearly coplanar to the benzo ring which favored a better packing. Upon complexation to BF_2_ moiety, three complexes 19 (n = 8, 10, 12) formed enantiotropic N or/and SmC phases. All clearing temperatures were higher than those of the precursor compounds. The presence of sulfur atom led to better polarization thereby resulting in better mesomorphic behavior and luminescence properties over the benzoxazole analogues too.

Recently, Cai et al. reported boron difluoride complexes 20 derived from aroylhydrazines that showed mesomorphic behavior and AIE properties (Cai et al., 2021). All precursor ligands of 20a–b were crystalline and non-mesogenic but all complexes 20 formed SmC or/and N phases. These BF_2_ complexes were fluorescent and the compound 20a was AIE active. As observed in single crystal of 20a (n = 8) the overall molecule was quite flat and a dihedral angle of ∼3.298° and ∼5.750° between the central core and the surrounding phenyl rings was observed. Weak intramolecular hydrogen bonds kept the central molecule as planar as possible making it easy for the molecules to pack in the crystal and liquid crystal states. Along with other intermolecular F···H interactions, formation of dimeric structures through hydrogen bonds along with other weak interactions further favored mesophase formation. Two derivatives 20a (n = 10) and 20b (n = 8) formed monotropic SmC phases and complexes 20b have a wider temperature range of SmC phase than that of complexes 20a that were attributed to more efficient π-π interactions between the cores upon naphthalene substitution. Compound 20a (n = 12) was found to be AIE active.

## Conclusion and Future Outlook

In this mini-review, we have summarized the recent developments in the supramolecular chemistry of tetra-coordinate boron-containing organic molecules. Organoboron compounds prepared from chelating units such as imines, diiminates, ketoiminates, diketonates, etc. are easy to synthesize and often afford good to excellent synthetic yields. As the chelating units are synthesized from simple starting materials like amines and aldehydes/ketones, this strategy allows for the synthesis of a variety of compounds whose properties may be tuned by an appropriate selection of the reactants. Further, the presence of heteroatoms and aromatic rings in these systems helps in stabilizing the self-assembled structures through various non-covalent interactions like H-bonding, CH-π and F-π interactions without the need for specific functionalization. As exemplified by various reports, a majority of these compounds exhibit excellent luminescence properties, and most of them have been used for sensing applications. However, a majority of these systems absorb UV or visible light and only limited success has been achieved in the synthesis of near-infrared absorbing dyes using this chemistry. NIR absorbing dyes are superior for biological applications and thus extension of this methodology for the synthesis of long-wavelength absorbing dyes would be beneficial for applications in biology. As these supramolecular assemblies are inherently dynamic and reversible, and have the ability to respond to external stimuli, a synergy between adaptive properties and NIR optical properties could lead to next-generation soft materials for biomedical applications. Another area with scope for improvement is the appropriate functionalization of organoboron compounds so as to obtain supramolecular hydrogels which will enable and extend the utility of these systems for wound healing, cell therapies, artificial tissue development and anticancer treatments. In order to be commercially successful, these compounds must be explored from the application perspective which in our opinion lacks to date. We hope that this mini-review gives a comprehensive understanding of the capabilities of the highly stable tetra-coordinate boron containing molecules to the readers and opens up new avenues towards real life applications.
